# Sensory trap leads to reliable communication without a shift in nonsexual responses to the model cue

**DOI:** 10.1093/beheco/arae006

**Published:** 2024-02-01

**Authors:** Skye D Fissette, Tyler J Buchinger, Sonam Tamrakar, Anne M Scott, Weiming Li

**Affiliations:** Department of Fisheries and Wildlife, Michigan State University, 13 Natural Resources Building, 480 Wilson Rd., East Lansing MI 48824, USA; Department of Fisheries and Wildlife, Michigan State University, 13 Natural Resources Building, 480 Wilson Rd., East Lansing MI 48824, USA; Department of Fisheries and Wildlife, Michigan State University, 13 Natural Resources Building, 480 Wilson Rd., East Lansing MI 48824, USA; Department of Fisheries and Wildlife, Michigan State University, 13 Natural Resources Building, 480 Wilson Rd., East Lansing MI 48824, USA; Department of Fisheries and Wildlife, Michigan State University, 13 Natural Resources Building, 480 Wilson Rd., East Lansing MI 48824, USA

**Keywords:** chemical ecology, receiver bias, sensory trap, signal evolution

## Abstract

The sensory trap model of signal evolution suggests that males manipulate females into mating using traits that mimic cues used in a nonsexual context. Despite much empirical support for sensory traps, little is known about how females evolve in response to these deceptive signals. Female sea lamprey (*Petromyzon marinus*) evolved to discriminate a male sex pheromone from the larval odor it mimics and orient only toward males during mate search. Larvae and males release the attractant 3-keto petromyzonol sulfate (3kPZS), but spawning females avoid larval odor using the pheromone antagonist, petromyzonol sulfate (PZS), which larvae but not males, release at higher rates than 3kPZS. We tested the hypothesis that migratory females also discriminate between larval odor and the male pheromone and orient only to larval odor during anadromous migration, when they navigate within spawning streams using larval odor before they begin mate search. In-stream behavioral assays revealed that, unlike spawning females, migratory females do not discriminate between mixtures of 3kPZS and PZS applied at ratios typical of larval versus male odorants. Our results indicate females discriminate between the sexual and nonsexual sources of 3kPZS during but not outside of mating and show sensory traps can lead to reliable sexual communication without females shifting their responses in the original context.

## Teaser

Males in some species signal to females using traits that mimic nonsexual cues, but how females evolve in response to being manipulated remains poorly understood. Using in-stream behavioral assays, we show that female sea lamprey use a mimetic male pheromone for reliable sexual communication, without shifting their response to the deceptive component in the nonsexual migratory context.

## INTRODUCTION

Many sexual signals exploit pre-existing responses that originated outside of the mating context (Ryan 1990; Christy 1995; Endler and Basolo 1998; Ryan and Cummings 2013). For example, male lyrebirds (*Menura novaehollandiae*) mimic alarm calls to prevent females from leaving courtship arenas (Dalziell et al. 2021), and male characin (*Corynopoma riisei*) have opercular ornaments that mimic ants and elicit foraging responses from females (Kolm et al. 2012). An understanding of mimetic signals such as these, known as sensory traps (West-Eberhard 1984; Christy 1995), helped to expand the discussion around signal evolution beyond classic models that suggest preferences for mating signals arise according to the reproductive benefits they provide or indicate (Andersson 1994; Møller and Jennions 2001; Hosken and House 2011). Despite much evidence that sensory traps underlie the origin of sexual signals, if and how receivers evolve in response to these deceptive signals remains largely unknown and subject to debate (Basolo 1996; Dawkins and Guilford 1996; Holland and Rice 1998; Arnqvist 2006; Rodríguez 2009; Ryan and Cummings 2013).

Some evidence indicates that sensory traps can lead to reliable sexual communication if receivers evolve to discriminate the mating signal from the nonsexual cue it mimics (Stuart-Fox 2005). In splitfin fishes (Goodeniae), males signal to females using a yellow tail band that mimics damselfly larva and invokes foraging responses in females (Garcia and Ramirez 2005). The deceptive signal imposes costs on females as they become less efficient at foraging when male tail bands distract them from actual prey (Garcia and Lemus 2012). These costs drove females to evolve a higher response threshold to the stimulus (a quivering yellow shape), which was then matched by exaggeration of the male tail band. The ensuing co-evolution of signal elaboration and resistance led females to decouple the male yellow band and damselfly larvae and stop responding to the stimulus as a feeding cue. However, as expression of the yellow band is costly, females seem to benefit from using it to assess male quality (Garcia and Ramirez 2005). These studies support the theory that exploitation of female preferences is costly to females and is met by resistance (Holland and Rice 1998; Arnqvist 2006) but show that this resistance can lead to reliable sexual communication (Stuart-Fox 2005).

Male sea lamprey (*Petromyzon marinus*) signal to sexually mature females using a sex pheromone thought to mimic the odor of conspecific larvae (Buchinger et al. 2013). During the pre-spawning migration into streams, sea lamprey follow chemical cues released by larvae that reside in nursery habitats near spawning grounds (Teeter 1980; Fissette et al. 2021). The larval odor consists of fecal metabolites including the bile acid 3-keto petromyzonol sulfate (3kPZS), which attracts migratory males and females upstream (Johnson et al. 2013; Brant et al. 2015, 2016). After several weeks in a stream, males become sexually mature (spermiated; Applegate 1950; Johnson et al. 2015), build nests on gravel bars, and signal to sexually mature (ovulated) females using a multi-component pheromone (Fissette et al. 2021). As with larval odor, the male pheromone consists, in part, of 3kPZS; males release 3kPZS at high rates (~0.25 mg/h) via specialized gill cells (Li et al. 2002; Siefkes et al. 2003) and ovulated females are attracted to 3kPZS over long distances (Siefkes et al. 2005; Johnson et al. 2009). Evidence that other lamprey species use 3kPZS as a component of larval odor but not a male pheromone indicates the nonsexual migratory response to 3kPZS may have originated before male signaling with 3kPZS (Buchinger et al. 2013, 2017). Taken together, these studies support the hypothesis that the male pheromone mimics the nonsexual larval cue.

**Figure 1:**
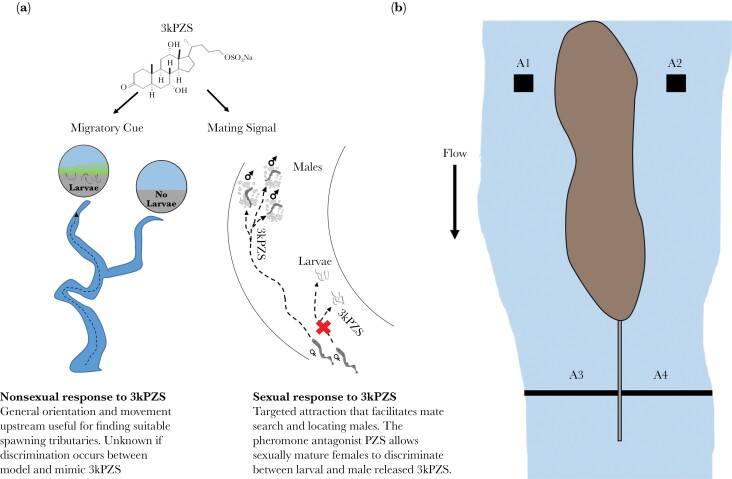
**Context dependent responses to 3kPZS and how they were tested within the nonsexual context of reproductive migration**. (A) The behavioral responses of female sea lamprey to 3-keto petromyzonol sulfate (3kPZS) are different within the contexts of migration and sexual reproduction. During reproductive migration, migratory sea lamprey display general orientation and upstream movement to larval released 3kPZS, which helps guide them to suitable spawning streams and tributaries within them (Johnson et al. 2013; Brant et al. 2016). During sexual reproduction, 3kPZS facilitates mate search, induces targeted attraction to a male’s nest, and helps ensure successful reproduction (Johnson et al. 2009), and PZS acts as a pheromone antagonist to allow ovulated females to discriminate between male and larval odor (Buchinger et al. 2020). (B) The upstream portion of the ~250 m experimental bioassay that was used to test whether migratory sea lamprey discriminate between the larval cue and its mimic in the nonsexual context. An island at the upstream end splits the river into two sub-channels and mimics a natural scenario of tributary selection. The length of the island was extended by ~8 m with a plywood wall that was tarped and lined with sandbags to ensure no odor mixing between channels. Individual lamprey were implanted with passive integrated transponder (PIT) tags, and their behavior was monitored using several PIT antennas throughout the experimental site. Antennas were used at two separate locations: 1) two antennas at the upstream end of each sub-channel where treatment odors were applied (A1 and A2) and 2) two stream width antennas just upstream of the channel choice point (A3 and A4).

Despite the deceptive origin of 3kPZS signaling, ovulated female sea lamprey evolved to use the male pheromone for reliable sexual communication (Buchinger et al. 2020). Sea lamprey die after a single spawning season, and ovulated females have only a few days to a week to find males and spawn (Applegate 1950; Johnson et al. 2015). Therefore, 3kPZS signaling conceivably benefits ovulated females by reducing the costs of mate search. However, ovulated females searching for mates encounter 3kPZS from both males and larvae, as larvae reside in habitats immediately downstream of and sometimes interspersed with spawning grounds. Consistent with the presumed costs of confusing larval odor (the model cue) and the male pheromone (the mimetic signal; see Dalziell and Welbergen 2016 for a general discussion on mimicry), ovulated females discriminate against larval odor and orient only toward the male pheromone during spawning (Buchinger et al. 2020). A mechanism underlying this discrimination is a pheromone antagonist, petromyzonol sulfate (PZS), which abates ovulated female preference for 3kPZS when mixed at equal or greater concentrations than 3kPZS and which larvae, but not males, release at higher rates than 3kPZS (Figure 1A; Buchinger et al. 2020). Discrimination of larval odor and male pheromone by ovulated females enables reliable sexual communication with 3kPZS, but it remains unknown if female responses have shifted in the nonsexual context as observed in splitfins (Garcia and Lemus 2012).

Here, we tested the hypothesis that migratory females discriminate their responses to the male pheromone and larval odor and orient only to larval odor in the nonsexual migratory context. Using in-stream behavioral assays, we determined responses of migratory females to 1) mixtures of synthesized 3kPZS and PZS at ratios typical of larval (1:10) and male (100:1) odorants and 2) the complete, natural odors of larvae and males. These experiments allowed us to test whether responses of migratory (nonsexual) females show the converse pattern of spawning (sexual) females, which would be expected if migratory females, as spawning females, presumably incur fitness costs by responding similarly to larval and male odor. Specifically, we tested the predictions that migratory females orient toward larval odor and larval-typical ratios of 3kPZS and PZS but not male pheromone or male-typical ratios of 3kPZS and PZS. Our results indicate females did not shift their responses to discriminate 3kPZS released by males versus larvae during migration as they did during spawning and support a previously undocumented evolutionary route to reliable sexual communication.

## METHODS

### Experimental animals

Sea lamprey were trapped in tributaries of Lake Huron from May-June by the United States Fish and Wildlife Service. Lamprey were then transported to United States Geological Survey, Hammond Bay Biological Station (HBBS), Millersburg, MI, USA and held in 1000 L aerated tanks that were continually supplied with Lake Huron water at ambient temperatures. All experimental procedures followed protocols approved by the Michigan State University Institutional Animal Care and Use Committee (PROTO202100029). Two days prior to experiments, a group of sexually immature females (*N* = ~250) was taken to the Ocqueoc River (Millersburg, MI, USA) and held in steel mesh cages to allow 1) acclimation to river conditions and 2) the natural sexual maturation process to remain ongoing in warmer river temperatures that are not available in holding conditions at HBBS (holding tanks contain much colder Lake Huron water). Every 2–3 days, additional migratory stage females (*N* = 75–150) were transported to holding cages in order to replace the females used in daily experimental trials (*N* = 45–54) and/or any individuals that died. The total number of migratory females held in cages ranged from 205 to 307 for the duration of the experiment. No sexually mature females were used during this experiment, and they were differentiated from migratory stage individuals by applying gentle pressure to the abdomen and checking for oocyte expression (Siefkes et al. 2003).

### Experimental procedures—passive integrated transponder tagging

For each trial, 15–18 migratory females were fitted with a 23-mm half-duplex passive integrated transponder (PIT) tag (Oregon RFID, Portland, OR, USA). In each female, a PIT tag was inserted via a small incision in the abdomen anterior to the first dorsal fin. After inserting the tag, the incision was closed using VetBond tissue adhesive (3M, St. Paul, Minnesota, USA). When tagging was complete, experimental subjects were held in 200 L aerated tanks until being taken to the Ocqueoc River to acclimate for ~20 h prior to the following day’s experimental trials.

### Experimental procedures—behavioral assays

In-stream assays were used to evaluate behavioral responses of migratory stage females to natural and synthesized odors released by larvae and sexually mature males, and because migrating sea lamprey are primarily nocturnal (Applegate 1950), behavioral experiments were conducted at night. Assays were conducted in a ~250 m stretch of the Upper Ocqueoc River from 29 May 2021 to 20 June 2021. This stretch of river contains an island at the upper reaches that splits the main channel into two sub-channels, and this island was further extended ~8 m with a plywood wall that was tarped and lined with sandbags to prevent mixture between sub-channels (Figure 1B). This bifurcation mimics a natural scenario faced during sea lamprey migration, where tributaries within river systems meet and create a decision point for lamprey, where they must select the proper tributaries to maximize opportunities for successful reproduction (Buchinger et al. 2015; Fissette et al. 2021). The Ocqueoc River is a historic spawning site (Applegate 1950), but currently, sea lamprey are blocked from the upper stretches by a barrier. This ensures no background pheromone odor from naturally spawning populations are present during experimental trials.

Migratory female behavior was monitored using a PIT array with four separate PIT antennas to determine distribution within the experimental assay during experimental trials (Figure 1B). Two antennas, one within each channel and spanning the entire stream channel width, were placed ~3–5 m upstream from the sub-channel confluence and were used to determine which sub-channel an individual entered. Two additional antennas (1 m^2^) were placed in each sub-channel at the upstream end of the island ~5 m below where the main river channel splits. Treatment odors were applied within the 1 m^2^ upstream PIT antennas. PIT antennas monitored the proportion of females entering each sub-channel. Females used during experiments were held in release cages ~250 m downstream from the odor application point. Three experimental trials were conducted each night ranging from ~21:40 to 01:15, with the first trial beginning 15 minutes after sunset. Each experimental trial was conducted for one hour. After 15 minutes of odor application, females were released from cages, and their behavior was monitored for 45 minutes. Odors were applied using a peristaltic pump (Masterflex L/S EW-07554-90, Cole Parmer, Vernon Hills, IL, USA). Unique PIT tag numbers were used for each individual and allow behavior to be analyzed only for the trial in which they were released.

To determine if migratory stage females discriminated between the larval and male odorants, we compared behavioral responses across seven different treatments that include synthetic (Bridge Organics Co., Vicksburg, MI, USA) and natural pheromone mixtures and were alternately applied to each sub-channel: 1) MeOH: water (1:1)—negative control, 2) larval extract (LE, migratory cue)—positive control, 3) spermiated male washings (SMW, male sex pheromone), 4) 3kPZS:PZS (1: 10), larval ratio, 5) 3kPZS:PZS (100: 1), male ratio, 6) 3kPZS:PZS (1: 10) versus 3kPZS:PZS (100: 1), and 7) LE versus SMW. Details on the natural odorant collection are described below. The negative control allowed the assessment of channel bias within the assay. The positive control (LE) replicated the whole migratory pheromone released by larvae and confirms the behavioral assay functions to capture the intended behaviors. Testing larval and male-released 3kPZS:PZS ratios is a direct test of whether migratory females discriminate between larval odor and the male pheromone during migration using the same mechanism (pheromone antagonist) as ovulated females. Individual ratios of 3kPZS:PZS showed whether migratory females are attracted to these different ratios on their own, and the application of these ratios simultaneously (3kPZS:PZS 100:1) versus (3kPZS:PZS 1:10), with one odor in each sub-channel, showed whether migratory females, like ovulated females, have a direct preference for either ratio. SMW applied alone showed if migratory stage females are attracted to the male sex pheromone, and the application of LE and SMW simultaneously (LE vs. SMW) showed if migratory females discriminate and prefer the natural larval or male pheromone.

To avoid confounding factors of channel bias and time of night, we applied each treatment to the left versus right sub-channels during approximately the same number of trials and distributed treatments across all experimental times of night (early, middle, and late trials). All treatments were applied to reach in-stream concentrations of 1.00E-13 M 3kPZS, which has been shown to induce behavioral responses in ovulated females (Johnson et al. 2009). Sub-channel discharges were taken every 2–3 days or after every rain event to calculate the amount of each odorant needed to reach desired concentrations. The 3kPZS:PZS ratios for synthetic odors (3kPZS:PZS, larva = 1: 10, male = 100:1) were chosen based on previous work (Buchinger et al. 2020). Variation in 3kPZS and PZS concentrations are expected in natural odorants released by males and larvae (Buchinger et al. 2020), so it is not unexpected that the ratios of 3kPZS:PZS in our natural odorant treatments (LE and SMW) were not identical to the synthetic ratios. However, they showed the same overall trends in compound concentrations, with more PZS than 3kPZS released by larvae (3kPZS:PZS, 1:3.74) and more 3kPZS than PZS released by sexually mature males (3kPZS:PZS, 40.3:1).

### Experimental procedures—natural pheromone collection

LE was collected by holding high densities of larval sea lamprey in large tanks at HBBS, and collection methods followed previously described methods (Li et al. 2018a). LE batches from five separate years were pooled together, mixed thoroughly, aliquoted into 1L bottles, and held at –80 °C until use. SMW was collected by holding 14 sexually mature males in 50 L of continually aerated Lake Huron water for 7 h. Males were then removed, the water thoroughly mixed, aliquoted into 1 L bottles and stored at –20 °C until use.

### Experimental procedures—chemical quantification

Quantification of chemical components present in LE and SMW was conducted using liquid chromatography tandem mass spectrometry (LC-MS/MS; Fissette et al. 2020) with slight modification. For LE samples, 1 mL aliquots were centrifuged at 12,500 *g* for 20 min at 4 °C (accuSpin Micro 17R, Fisher Scientific, Hampton, NH, USA). The supernatant was collected and filtered through 0.22 µm PVDF membrane filter (Millex ® - GV, Merck Millipore Ltd., Ireland). Prior to the LC-MS/MS analysis, the filtrate was diluted with an equal volume of methanol (1:1, v/v). For SMW samples, 10 mL aliquots were freeze dried. The residue was dissolved in 1 mL methanol and centrifuged at 12,500 *g* for 20 min at 4 °C. The supernatant was freeze dried and reconstituted in 100 µL of 50% methanol in water for LC-MS/MS analysis. Samples were analyzed on a ACQUITY H-Class UPLC™ coupled to a Xevo TQ-S triple quadrupole mass spectrometer (both Waters Corporation, Milford, MA, USA). A BEH C18 column (2.1 × 100 mm, 1.7 µm particle size, 130 Å; Waters Corporation) was used with 10 mM trimethylamine (TEA) in water as mobile phase A, and methanol as mobile phase B. The injection volume was 10 µL, and the HPLC flow rate was 0.25 mL/min. Mass spectra were acquired using multiple reaction monitoring (MRM) mode with electrospray ionization in negative ion mode. MassLynx 4.2 software was used for data acquisition, and data were processed using TargetLynx XS (Waters Corporation).

### Statistical analyses

All statistical analyses were conducted in R v3.5.1 (Team 2018). The proportions of migratory females entering each sub-channel were analyzed using a mixed-effects logistic regression model with a binomial distribution. A separate model was run for each treatment, and all models evaluated the effect of odor on which sub-channel a migratory female entered and tested for channel bias. All analyses used the lme4 (Bates et al. 2015) and car (Fox et al. 2012) packages with type III sums of squares (α = 0.05). For all treatments, only migratory females that swam upstream from the release point and entered a sub-channel were included in statistical analyses: (MeOH: water (1:1), 29/128 (23%); LE, 58/129 (45%); SMW 49/133 (37%); 4) 3kPZS:PZS (1: 10), 32/125 (26%); 3kPZS:PZS (100: 1), 43/122 (35%); 3kPZS:PZS (1: 10) versus 3kPZS:PZS (100: 1), 31/140 (22%); LE versus SMW, 51/111 (46%). For the negative control, a “treatment” side was randomly assigned for the first trial and alternated across subsequent trials.

## RESULTS

### Migratory females were attracted to both larval and male 3kPZS:PZS ratios and did not discriminate between them

**Figure 2:**
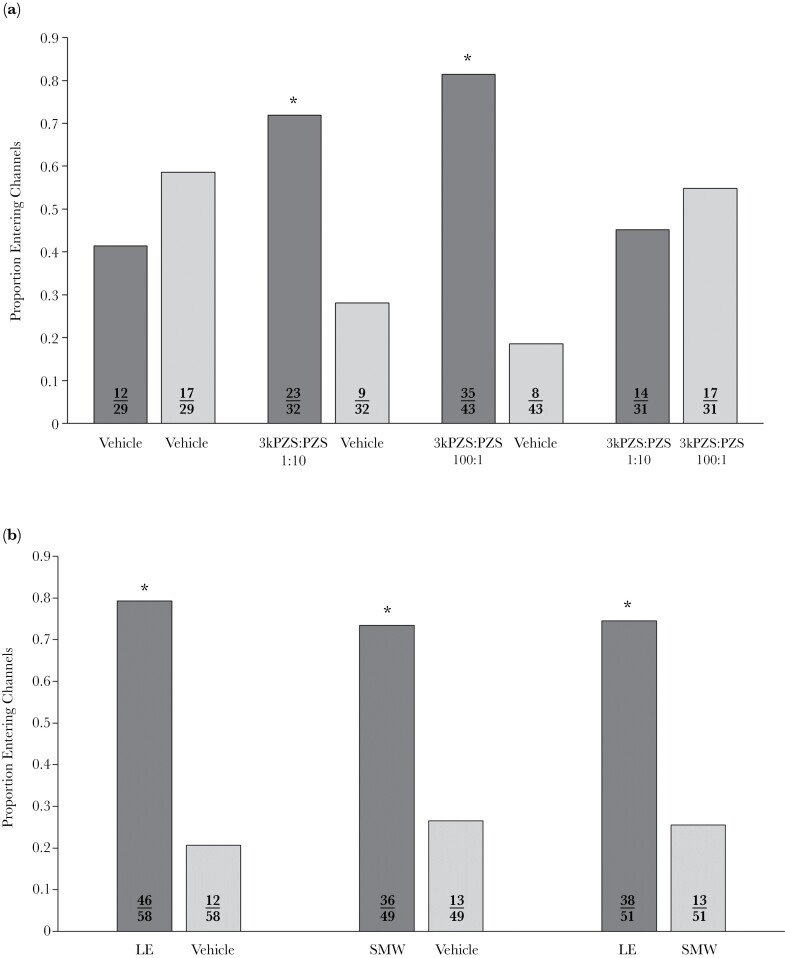
**Behavioral responses of migratory female sea lamprey to mixtures of natural and synthetic odorants.** Bar charts display the proportion of migratory individuals entering each sub-channel during behavioral experiments. (A) The synthetic pheromone mixtures tested were a mixture of 3-keto petromyzonol sulfate (3kPZS) and petromoyzonol sulfate (PZS). These were tested at the larval released ratio (3kPZS:PZS, 1:10) and male-released ratio (3kPZS:PZS 100:1) as previously reported (Buchinger et al. 2020). A negative control, with the vehicle (MeOH: water (1:1) in each sub-channel was also tested. (B) Natural odor mixtures tested were the migratory pheromone released by sea lampreBuchinger et al. 2020y larvae (LE) and the mating pheromone released by males (SMW). All treatments were standardized by 3kPZS application to reach in-stream 3kPZS concentrations of 1.00E-13M. Separate mixed-effects logistic regression models with a binomial distribution were used to analyze each treatment in order to assess how different natural or synthetic pheromone mixtures influence attraction via channel selection. Asterisks indicate *P* < 0.05, and the numbers within each bar represent the proportion of migratory females entering each sub-channel over the duration of the experiment.

Migratory females showed no preference for either sub-channel during negative control trials (χ² (1) = 0.47, *P* = 0.49, Figure 2A). When odors were tested individually, migratory females were attracted to 3kPZS:PZS mixtures at the ratio released by larvae (1:10) (χ² (1) = 8.01, *P* < 0.01, Figure 2A) and released by males (100:1), (χ² (1) = 41.9, *P* < 0.001, Figure 2A), and they showed no preference when exposed to both ratios of 3kPZS:PZS mixtures simultaneously (χ² (1) = 1.59, *P* = 0.21, Figure 2A). A channel bias was observed during 3kPZS:PZS (100:1) trials (χ² (1) = 24.7, *P* < 0.001) but not for 3kPZS:PZS (1:10) (χ² (1) = 2.17, *P* = 0.14), direct comparison (100:1 vs. 1:10) (χ² (1) = 2.90, *P* = 0.09), or negative control (χ² (1) = 0.47, *P* = 0.49) trials.

### Migratory females were attracted to both the larval and male odor individually but do discriminate between them

Migratory females were attracted to both the larval cue (LE) (χ² (1) = 56.5, *P* < 0.001, Figure 2B) and the male sex pheromone (SMW) (χ² (1) = 40.5, *P* < 0.001, Figure 2B) but when individuals were exposed to both simultaneously, with a different odor in each sub-channel, migratory females preferred the sub-channel baited with LE over the sub-channel baited with SMW, (χ² (1) = 6.51, *P* = 0.01, Figure 2B). A channel bias was observed during LE (χ² (1) = 35.9, *P* < 0.001,) and SMW (χ² (1) = 29.8, *P* < 0.001) trials but not during direct comparison trials (LE vs. SMW) (χ² (1) = 0.96, *P* = 0.33).

## DISCUSSION

Many animal courtship signals appear to mimic nonsexual cues (Ryan and Cummings 2013); nevertheless, our knowledge of how these so-called “sensory traps” shape the evolution of communication remains incomplete due to limited data on how receivers respond to manipulation by signalers. To better understand how receivers might adapt to sensory traps, we tested the nonsexual behavioral responses of migratory female sea lamprey to the deceptive component of a male sex pheromone suspected to mimic a cue for productive larval habitat (Buchinger et al. 2013). Previous evidence indicated that, when searching for mates, spawning female sea lamprey use the pheromone antagonist PZS to discriminate between the larval and male odorants despite each containing the attractant 3kPZS (Buchinger et al. 2020). In the current study, behavioral assays in a natural stream showed that migratory females, which have not yet begun to search for mates, move upstream toward 3kPZS when it was mixed with PZS at ratios typical of males or larvae and, furthermore, did not discriminate between mixtures at either ratio. Our results indicate females have not evolved to discriminate between 3kPZS released by males versus larvae during the pre-spawning migration and suggest a previously undocumented evolutionary trajectory of a sensory trap in which females use a mimetic male signal for reliable sexual communication without changing their responses to the original nonsexual cue.

Our results support suggestions that courtship signals exploiting nonsexual responses need not be costly to receivers (Christy 1995; Ryan and Cummings 2013). Discussions on evolution of communication via sensory traps or other perceptual biases often emphasize the costs they bring to receivers (Arnqvist 2006), which might arise from sub-optimal matings (Toft and Albo 2015; Martínez Villar et al. 2021) or functional disruption of the response to the nonsexual cue (Bradbury and Vehrencamp 2000; Garcia and Lemus 2012). However, though these signals are inherently deceptive, sensory traps may still benefit receivers (Christy 1995). In sea lamprey, as other animals (Ryan and Cummings 2013), an obvious potential benefit of the mimetic male signal is reduced mate search costs. A signal that hastens mate search is likely beneficial to ovulated female lamprey as they have limited time (a few days to a week; Applegate 1950; Johnson et al. 2015) and energy reserves (William and Beamish 1979) to find nesting males and spawn before they die. Indeed, evidence that female sea lamprey have adapted to use 3kPZS as a sexual signal indicates it benefits them during mating (Buchinger et al. 2013, 2020). Likewise, the current study implies that male signaling with a deceptive pheromone does not impose costs on females outside of mating; migratory females oriented toward natural pheromone from both larvae and males and did not discriminate between larva- and male-typical ratios of synthesized 3kPZS and PZS (see below regarding the observed discrimination between natural male and larval odorant). These results contrast with those from studies on spawning females, which likely incur costs when responding similarly to larval and male odorant and have evolved to discriminate between the two using the ratio of 3kPZS and its antagonist PZS (Buchinger et al. 2020). Responding similarly to 3kPZS within the odor of males versus larvae during migration makes sense for at least two reasons: first, males are unlikely to use 3kPZS to coerce females into sub-optimal mating because migratory females are not physiologically capable of spawning (Docker et al. 2019) and their behavioral response to 3kPZS during migration brings them upstream but not to the source of pheromone release (i.e., a male; Buchinger et al. 2013; Brant et al. 2015, 2016). Second, the odors of both larvae and males provide migratory females with information relevant to their migratory decisions. Larval odor attracts females into habitat suitable for offspring success (Wagner et al. 2009; Buchinger et al. 2015), whereas male pheromone could still bring females to appropriate spawning tributaries. In contrast, signals that mimic food (Herzner et al. 2005) or predators (Nakano et al. 2013) seem less likely to provide females with information relevant to foraging or predator avoidance. Notably, some mimetic signals provide benefits to receivers without having any link to reproduction, such as the structures built by male fiddler crabs that provide females cover from predators (Christy et al. 2003). As most research on perceptual biases has focused on signals that mimic cues related to food and predation (Ryan and Cummings 2013), a more complete picture of how these processes shape the evolution of communication will likely require focus on a more diverse set of nonsexual responses that might be associated with different levels of fitness costs (or benefits) to exploited receivers.

We postulate that any costs male exploitation might bring females are further limited by the male pheromone being only a partial mimic of larval odor. Mimicry is expected to be imperfect and only requires receivers to perceive a mimic and its model as similar (Dalziell et al. 2021). Evidence that the male pheromone mimics larval odor comes from observations that 3kPZS is released by both males and larvae (Li et al. 2002; Brant et al. 2016), attracts females and males outside of spawning (Johnson et al. 2013; Brant et al. 2015), and elicits nonsexual responses in lamprey species that do not use it as a sex pheromone (Buchinger et al. 2013, 2017). However, both the larval cue and male pheromone are multi-component mixtures (Sorensen et al. 2005; Johnson et al. 2012; Li et al. 2018a, 2018b) that only partially overlap in chemical composition (Buchinger et al. 2015; Fissette et al. 2021). Our observation that migratory females preferred the natural odor of larvae over males when each were applied at 1 × 10^–13^ M 3kPZS indicates the male pheromone is only a partial mimic of larval odor, with components not present in male odor guiding migratory females to larval odor. As outlined above, responding similarly to male and larval odors during migration seems unlikely to be costly to females; however, that migratory females can discern between the male pheromone and larval odor may further weaken any selection for females to change their nonsexual responses to 3kPZS. We suggest that the extent to which mimetic signals match their model cues is likely to influence the evolutionary trajectory of sensory traps and speculate that many sensory traps might not impose significant costs on receivers because they are imperfect mimics.

An important caveat to our study is that we only tested PZS as the mechanism by which females could specifically discriminate 3kPZS released by males versus larvae. We focused on PZS because it is structurally similar to 3kPZS (differing only by two hydrogens), interacts with at least some of the same receptors with 3kPZS (Siefkes and Li 2004; Zhang et al. 2020), and, most importantly, is a key mechanism by which spawning female lamprey distinguish between 3kPZS within larval odor versus male odor (Buchinger et al. 2020). We interpret this as evidence females have not shifted their responses to the deceptive component 3kPZS to allow them to respond differently to it when released within male versus larval odor. However, an alternative possibility is that migratory females evolved to distinguish between male- and larva-released 3kPZS using a molecule other than PZS. Indeed, we observed that migratory females prefer the natural odor of larvae over males, though we suspect this is due to the additional components of larval odor that are attractive (Sorensen et al. 2005; Li et al. 2018a) rather than the presence of molecules that specifically influence responses to 3kPZS, as does PZS (Buchinger et al. 2020). Regardless, our results clearly show migratory females orient toward the male pheromone in the nonsexual migratory context and provide strong evidence that the deceptive component 3kPZS remains coupled between the male pheromone and larval odor.

In summary, we provide evidence that a sensory trap led to reliable sexual communication without receivers shifting their nonsexual responses to the stimuli shared between the model and mimic. Female sea lamprey have adapted to use a mimetic male pheromone for effective mate search (Buchinger et al. 2020) but continue to respond to both the male pheromone and larval odor during migration and do not discriminate the deceptive component 3kPZS from larval versus male odor. Taken together, studies on if and how female sea lamprey respond to male pheromone and larval cue in contexts of migration and spawning illustrate how receivers can evolve context-appropriate responses mimetic sexual signals. The evidence from sea lamprey and splitfins suggests two distinct evolutionary routes by which sensory traps can lead to reliable sexual communication and points to a need for work on other species to develop a general framework of how these and other perceptual biases influence not just the origin but also the subsequent evolution of communication.

## Data Availability

Analyses reported in this article can be reproduced using the data provided by Fissette et al. (2024).
